# Effects of Pregnancy and Lactation on Bone Microstructure and Material Properties in a Rat Model of Bariatric Surgery

**DOI:** 10.1007/s00223-024-01321-1

**Published:** 2025-01-04

**Authors:** Malory Couchot, Françoise Schmitt, Morgane Mermet, Céline Fassot, Guillaume Mabilleau

**Affiliations:** 1 Univ Angers, Nantes Université, ONIRIS, Inserm, RMeS, UMR 1229, 49000 Angers, France; 2 Univ Angers, HIFIH, 49000 Angers, France; 3 Paediatric Surgery Department, CHU Angers, 49933 Angers, France; 4 Univ Angers, Inserm, CNRS, MITOVASC, 49000 Angers, France; 5 Cell and Tissue Pathology, CHU Angers, 49933 Angers, France

**Keywords:** Vertical sleeve gastrectomy, Bone mass, Bone microarchitecture, Bone material properties, Pregnancy, Lactation

## Abstract

Obesity is a major public health issue worldwide. Despite various approaches to weight loss, the most effective technique for reducing obesity, as well as diabetes and associated diseases, is bariatric surgery. Increasingly, young women without children are undergoing bariatric surgery, vertical sleeve gastrectomy (VSG) being the most common procedure nowadays. However, despite several reports suggesting bone loss after VSG, little is known about the potential additive effects of gestation and lactation after VSG to bone health. This study investigated the combined effects of pre-gestational VSG and subsequent gestation/lactation on bone metabolism in a rat model fed a high fat high sugar (HFHS) diet, with a focus on bone biomechanics, mass, microarchitecture and material properties. Furthermore, bone mass and remodelling were followed longitudinally by microCT prior to surgery, 4 weeks post-surgery, after weaning and at sacrifice. Significant alterations in bone mass and microarchitecture, characterized by changes in trabecular thickness and number, as well as changes in bone formation and resorption were influenced by both surgery and reproductive demands. Mechanical testing at sacrifice demonstrated compromised long bone fragility, in rat with HFHS regardless of the surgical procedure (Sham or VSG). Furthermore, analysis of bone material properties highlighted potential disruptions in the pattern of bone mineralization in sham and VSG animals fed a HFHS diet. These findings underscore the complex interplay between pre-gestational VSG and subsequent gestation/lactation in modulating bone metabolism in the investigated rat model. The preclinical rat model may help with optimizing surgical strategies and developing targeted interventions to mitigate potential bone-related complications associated with VSG in reproductive-aged individuals.

**Supplementary Information:**

The online version contains supplementary material available at 10.1007/s00223-024-01321-1.

## Introduction

Obesity is a growing epidemic worldwide, with numerous health complications and associated problems including bone frailty [1]. Obesity is defined as a body mass index (BMI) ≥ 30 and is thought to affect nowadays around 890 million adult individuals and 160 million children worldwide [2]. Bone fragility is thought to occur in children, but it is less clear whether this also happens in adult individuals [3]. Although excessive weight can augment bone mass and mineral density, it also increases the risk of fracture at specific sites [4].

The current management of obese individuals is represented by lifestyle changes and therapeutic monitoring, accompanied by an increase in physical activity [5]. Pharmacological treatment may also be prescribed, such as orlistat, bupropion with naltrexone, phentermine with topiramate, lorcaserin, glucagon-like peptide-1 (GLP-1) receptor agonists or dual glucose insulinotropic polypeptide receptor/GLP-1 agonists [6].

For individuals struggling with severe obesity associated with comorbidities, bariatric surgery is currently the best option to lose weight and improve overall health. There are numerous types of bariatric surgeries, some being restrictive, others malabsorptive, and some combining both approaches. Today, the most performed type of bariatric surgery is vertical sleeve gastrectomy (VSG—restrictive) accounting for 60.4% of all the surgeries performed worldwide [7]. Following VSG, where most of the stomach is surgically resected, there are deficiencies in micronutrient absorption such as iron, zinc, selenium, calcium, vitamin D, folate and B12 [8]. Deficiencies in folate and vitamin B12 have previously been linked to elevated homocysteine levels [9], that can cause collagen cross-linking impairment [10], alteration of osteoclast and osteoblast activities [11, 12], leading to poor bone health and fragility fracture [13]. Previous research involving humans and rodents has shown that VSG leads to rapid bone loss and an elevated risk of osteoporosis and is associated with a twofold increase in fracture risk over time [14–17]. When examining the specific sites where fracture risk is increased, it is mainly at the wrist, hip, and femoral neck [17]. Initially, this bone loss was linked to vitamin D deficiency and reduced calcium absorption [18]. However, later studies revealed that even with sufficient vitamin D levels, VSG leads to increased markers of bone turnover, heightened marrow adiposity, and other significant effects on bone metabolism [19]. Despite these findings, the mechanisms by which VSG affects bone health independently of calcium and vitamin D are not well understood in humans. Li et al. also reported an elevation of granulocyte-colony stimulating factor (G-CSF) following VSG, responsible for changes on the bone marrow niche [20]. However, a recent study using subcutaneous administration of a monoclonal antibody targeting the GSFR, failed to demonstrate positive effects on bone strength despite clear improvement in trabecular bone mass [21]. The same observations were seen with a monoclonal antibody targeting sclerostin although with a lower efficacy in restoring trabecular bone mass [21]. This suggest that the bone fragility observed following VSG might not be fully associated to bone loss. An important contributor of bone strength, that has been poorly been investigated in the context of VSG, is represented by bone material properties [22]. Bone material properties encompass a series of parameters describing the composition of the bone extracellular matrix, including post-translational modifications of bone collagen and structural organization of the bone mineral [23]. As such, it appears important to investigate bone material properties following VSG.

As obesity is a known factor of infertility [24], there is an increasing demand of obese women of child-bearing age to undergo bariatric surgery and VSG in order to restore fertility [25]. Pregnancy and lactation periods are known factors affecting skeletal health of mother with a reversible trabecular and cortical bone loss due to changes in hormonal regulation associated with alterations of bone material properties [26–28]. However, little is known on the skeletal health of mother that prior to pregnancy underwent a VSG and especially whether the pregnancy/lactation periods could additively aggravate the compromised bone phenotype and fragility after VSG.

The aim of this study was therefore to develop a rat model of vertical sleeve gastrectomy followed by pregnancy and lactation periods to study their effects on skeletal health. A longitudinal follow-up by in vivo microCT of the trabecular and cortical bone mass at the tibia, as well as a longitudinal evaluation of bone remodelling was performed. At sacrifice, bone pieces were further recovered and a full evaluation of bone biomechanic, mass, microarchitecture and material properties were done.

## Materials & Methods

### Animals

Sprague–Dawley females aged 6-week-old were fed with a high fat high sugar (HFHS) diet composed of high fat pellets (824,053, Special Diet Services, Witham, UK) and sweetened condensed milk (Nestlé, Issy-les-Moulineaux, France) for 8 weeks. Supplementary Fig. 1 recapitulates the procedures sustained by all animal groups. Abdominal adipose tissue percentage was increased significantly at 14 weeks of age in HFHS-fed animals suggesting that the diet was effective (Supplementary Fig. 2). At 14 weeks of age animals were randomly allocated to one of the surgery groups represented by either sham (HFHS-Sham group, *n* = 7), or vertical sleeve gastrectomy (HFHS-VSG group, *n* = 10). Surgery was performed under gaseous anesthesia with isoflurane supplemented with buprenorphine (0.2 mg/kg bw). The VSG procedure was inspired by the methodology proposed by Ayer et al. and adapted to rat animals [29]. Briefly, the VSG procedure consisted in discarding 2/3 of the stomach by performing a median laparotomy with a midline incision spreading from the sternum to the middle of the abdomen taking care not to damage the abdominal muscles. The short gastric and right gastro-epiploic vessels were ligated using 6/0 absorbable sutures. The rumen and the fundus were then resected, with blood loss controlled using, when required, a high temperature sterile cautery pen (#F7244, Fiab, Firenze, Italy). The stomach was then closed with a linear 6/0 absorbable suture and repositioned in its anatomical position into the abdomen. The abdominal wall was closed using V-loc™90 absorbable sutures (Covidien, France) in two layers. The Sham procedure consisted in performing a median laparotomy with a midline incision spreading from the sternum to the middle abdomen taking care not to damage the abdominal muscles. No organ resection was then performed. The abdominal wall was closed using V-loc™90 absorbable sutures in two layers. From three days before surgeries, animals were given vitamin supplements in drinking water (vitaRongeur, Virbac, Carros, France) for a period of 6 days. As previously reported by Kruger and Morel, Sham operation does not impact bone metabolism as compared with non-operated animals [30]. During the 72 h post-surgery, animals received subcutaneous administrations of buprenorphine (0.2 mg/kg bw) and amoxicillin/clavulanic acid (50 mg/kg/day—5 mg/kg/day). Eight age- and sex-matched Sprague–Dawley rats under normal rodent diet (A04, Safe, Augy, France) and sham-operated were used as controls (SD-Sham group). These animals received also the vitamin supplements given to HFHS-fed animals.

At 18 weeks of age, all animals were mated with Sprague–Dawley male rats and only operated-females that had offspring were sacrificed at 30 weeks of age. Animals were housed in social groups and maintained in a 12 h:12 h light:dark cycle and had free access to water and diet ad libitum. All animals received intraperitoneal administrations of calcein green (10 mg/kg) 10 days and 2 days before sacrifice under gaseous anesthesia. Bone pieces were collected at the time of sacrifice for further analyses.

### In Vivo Microcomputed Tomography (In Vivo microCT)

A longitudinal follow-up of bone microstructure at the proximal metaphysis of the right tibia was performed by in vivo microCT under general gaseous anaesthesia before surgery, and 4-,10- and 16-week post-surgery. Briefly, the proximal metaphysis was analysed with a Bruker 1076 microtomograph (Bruker Skyscan, Kontich) operated at 80 kV and 120 µA. An isotropic voxel size of 18 μm was used with an integration time of 2000 ms and a rotation step of 0.5° using a 1-mm aluminium filter. Reconstruction of 2D projections was done using NRecon software (version 1.6.10.2) using ring artefact correction set at 10, beam hardening correction set at 25% and a median filter. For trabecular analysis, a volume of interest located 0.5 mm below the growth plate and extending 2 mm down was chosen. For cortical analysis, a volume of interest (0.5 mm) centred 3 mm below the growth plate was chosen. Global threshold set at 300 mg/cm^3^ and 700 mg/cm^3^ hydroxyapatite were used for segmenting trabecular and cortical bone tissues, respectively. Between two microCT scans, regions of interest (ROI) were automatically aligned with the dataviewer software (version 1.5.6.2) using morphometrical features. Volumes of bone formation and resorption were computed with a lab-made Matlab script (Matlab R2021b, The Mathworks, Natick, MA) allowing for longitudinal evaluation of bone remodelling at the proximal metaphysis. Briefly, the two datasets were registered, and voxels present only in the older dataset were assigned to bone resorption. Voxels present only in the younger dataset were assigned to bone formation. Bone formation and bone resorption were computed as the sum of unique voxels divided by the sum of common and unique voxels. All bone histomorphometrical parameters were measured with the CTan software according to guidelines and nomenclature proposed by the American Society for Bone and Mineral Research.

For determination of abdominal adipose tissue at 14 weeks of age, the pre-surgery dataset was reconstructed in NRecon software as described above. Hip bones were detected as anatomical landmarks, and the volume of interest extended 100 sections above. The volume of interest was further masked to exclude skin and subcutaneous fat tissue. In parallel air and water standards were scanned with the same parameters and used to calibrate the X-ray CT density in Hounsfield units (HU). Adipose tissue was detected on each tomogram section as voxels with HU comprised between −280 and −150. The outer perimeter of the abdominal cavity was used as the peripheral border of the tissue volume and was filled in. The percentage of abdominal adipose tissue was computed as the sum of all voxels of adipose tissue divided by the sum of all tissue voxels.

### Ex Vivo microCT

Right tibia and femur (after 3-point bending) were analysed hydrated ex vivo by microCT with a Bruker 1272 microtomograph at 70 kv, 140 µA, 2000 ms integration time, an isotropic voxel size fixed at 7 μm, a rotation step set at 0.5°, and the use of a 1-mm aluminium filter. Hydroxyapatite phantoms (250 mg/cm^3^ and 750 mg/cm^3^) were used for calibration. Reconstruction of 2D projections was done using the NRecon software using ring artefact correction set at 10, beam hardening correction set at 25% and a median filter. Datasets were re-axed on the sagittal and coronal planes using the dataviewer software and transaxial sections (tibia and femur) were saved and further analysed with the CTan software. In order to analysed trabecular bone at the proximal tibia metaphysis and distal femur metaphysis, volumes of interest located 0.5 mm below/above the growth plate and extending 2 mm down/up were chosen. Trabecular bone was separated from cortical bone with an automatic contouring script in CTan software. Cortical analysis was restricted to tibia and femur, and the analyzed ROI (0.5 mm in length) was centred at 3 mm under/above the growth plate. Bone was segmented from soft tissue using global thresholding set at 300 mg/cm^3^ for trabecular bone and 700 mg/cm^3^ for cortical bone. All histomorphometrical parameters were measured with the CTan software according to guidelines and nomenclature proposed by the American Society for Bone and Mineral Research.

### Bone Biomechanical Test

Bending strength was measured by 3-point bending as described previously [31] and in accordance with published guidelines [32] using a constant span length of 15 mm. Right femurs were tested in the anteroposterior axis with the posterior surface facing upward, centred on the support, and the pressing force was applied vertically to the midshaft of the bone. Each bone was tested fully hydrated and at room temperature with a loading speed of 0.5 mm.min^−1^ until failure with a 500-N load cell on an Instron 5942 device (Instron, Elancourt, France), and the load–displacement curve was recorded at a rate of 100 Hz using Bluehill 3 software (Instron). Ultimate load, ultimate displacement, stiffness, and work-to-fracture were calculated as indicated in Turner and Burr [33]. The yield load was determined as the point at which a regression line that represents a 10% loss in stiffness crossed the load–displacement curve. Post-yield displacement was computed as the displacement between yielding and fracture. Additionally, tissue strength was calculated as the peak moment bending divided by the section modulus as reported in [34].

### Raman Spectroscopy

After 3-point bending, the proximal part of the right femur was embedded undecalcified in polymethymethacrylate (pMMA) at 4 °C as previously reported [35]. Thick bone Sects. (1 mm-thickness) were cut at the diaphyseal side, and the section surface was then grinded with sandpaper and polished with diamond paste (Struers, Champ sur Marne, France). In order to account for tissue age by using double calcein labellings, sections were imaged with a laser scanning confocal microscope (Leica DM8, Leica microsystems, Nanterre) set with an excitation light at 488 nm and an emission range 515–550 nm. Spectra were acquired using a Renishaw inVia Qontor confocal Raman microscope equipped with a 785 nm line laser, and a charged-coupled device (CCD) array detector. The laser power was set at 30 mW. The laser beam was focused onto the sample surface through an objective of 20 X and 0.4 numerical aperture, providing a spatial resolution of 2.4 µm and limiting polarization artefacts. Prior to each scan, spectral calibration was done using an internal silicon standard. The spectral range was set between 800 and 1800 cm^−1^, the acquisition time at 20 s and 3 accumulations. All data acquisition was performed using Renishaw WiRE v.5.5 software. Spectral artefacts including cosmic rays and background were removed using the nearest neighbour method and polynomial fit, respectively. Contribution of the embedding resin was subtracted using the major peak located at ~ 812 cm^−1^. Bone material parameters were: mineral-to-matrix ratio (intensity ratio between v1PO_4_ at ~ 960 cm^−1^ and Amide I at ~ 1668 cm^−1^), carbonate/phosphate ratio (intensity v3CO_3_ at ~ 1070 cm^−1^ and v1PO_4_), nanoporosity, computed before pMMA subtraction (intensity ratio between pMMA at ~ 812 cm^−1^ and v1PO_4_), mineral crystallinity (as the inverse of full width at half maximum of the v1PO_4_), proline hydroxylation content (intensity ratio between hydroxyproline at ~ 872 cm^−1^ and proline backbone at ~ 854 cm^−1^), glycosaminoglycan content (intensity ratio between GAG at ~ 1375 cm^−1^ and Amide III at ~ 1245 cm^−1^), collagen maturity (intensity ratio between ~ 1670 cm^−1^ and ~ 1690 cm^−1^).

Spectra were acquired in two different locations: (I) to investigate bone material properties at site of bone formation, spectra were randomly positioned between double calcein labellings and acquired as indicated above, and (II) to investigate bone material properties on the full cortical width, a line of points was drawn on the anterior quadrant extending from the periosteal to endosteal surfaces with a spatial resolution of 6 µm and acquired as above. For the later, histogram distribution for each compositional parameters were fitted with a Gaussian model and considered normally distributed if the R^2^ coefficient was > 0.95. In the present study, no histogram deviated from normal distribution. For each of the compositional parameters, the mean of the pixel distribution (excluding non-bone pixels) was computed as:

$$Mean = \sum \frac{{X_{i} \times F_{i} }}{N}$$

With Xi, representing the value of the compositional parameter for the i^th^ bin, Fi, representing the percentage of bone area with the Xi value and N, representing the total number of bins in the histogram distribution. The full width of the half maximum of each distribution was also computed to determine heterogeneity in the compositional parameter.

### Statistical Analysis

Statistical analyses were performed with GraphPad Prism 8.0 (GraphPad Software, La Jolla, CA, USA). The Brown-Forsythe test of equal variance was applied, and when normality was respected, ordinary one-way ANOVA with multiple post hoc Dunnett comparisons were conducted. When normality was not respected, Kruskal–Wallis tests with multiple post hoc Dunn’s comparisons were carried out. A linear regression was calculated to establish a correlation between the results of the in vivo microCT study and those of the ex vivo microCT study. Differences at *p* < 0.05 were considered significant.

## Results

### Longitudinal Evaluation of Bone Mass and Remodelling at the Proximal Tibia Metaphysis

**Fig. 1:**
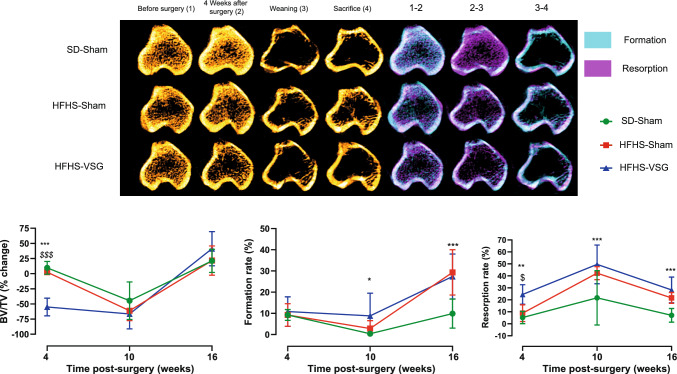
In vivo follow-up investigation of trabecular bone mass and remodelling at the proximal tibia metaphysis. MicroCT images taken before surgery (1), 4 weeks after surgery (2), at weaning (3) and at sacrifice (4). Bone formation and resorption rates were computed before and 4 weeks after surgery (period 1–2), between 4 weeks post-surgery and weaning (period 2–3), and between weaning and sacrifice (period 3–4). Quantitative analysis of BV/TV is reported as % change from previous time l. Statistical differences between SD-Sham (*n* = 8), HFHS-Sham (*n* = 7) and HFHS-VSG (*n* = 10) were computed using a one-way ANOVA with Tukey multiple comparisons. *: *p* < 0.05; **: *p* < 0.001; ***: *p* < 0.0001 vs. SD-Sham and ^$^: *p* < 0.05; ^$$$^: *p* < 0.0001 vs. HFHS-Sham at each time point

The right tibia of all animals was scanned just before surgery, 4 weeks after surgery, 10 weeks post-surgery, corresponding to weaning, and 16 weeks post-surgery at sacrifice. Interestingly, in the SD + Sham group, animals sustained a trabecular bone loss by ~ −54% (*p* < 0.001) at weaning but quickly gained trabecular bone mass post-weaning to reach a ~ 66% (*p* < 0.001) augmentation in this parameter at sacrifice (Fig. 1). These fluctuations of bone mass were accompanied by modifications of the pattern of bone formation with a significant decrease in trabecular bone formation by 9% (*p* < 0.001) at weaning and a significant increase by 10% at sacrifice. No significant modification of the pattern of bone resorption was observed in this group. In the HFHS-Sham group, a significant 64% decrease (*p* < 0.001) in BV/TV was observed at weaning and accompanied by significant reduction in bone formation (−6%, *p* = 0.017) and augmentation in bone resorption (+ 33%, *p* < 0.001). At sacrifice, all HFHS-Sham animals gained BV/TV by ~ 83% (*p* < 0.001) due to increase in bone formation (+ 26%, *p* = 0.023) and decrease in bone resorption (−21%, *p* = 0.024). In the HFHS-VSG group, in opposition to HFHS-Sham and SD-Sham, a significant loss of BV/TV by approximately 55% (*p* < 0.001) was evidenced 4 weeks post-surgery. Interestingly, these animals did not lose further bone at weaning (*p* = 0.50). However, during the weaning-sacrifice period, these animals presented with the largest gain in BV/TV (+ 75%, *p* < 0.001) associated with a 19% (*p* = 0.003) augmentation in bone formation and a 22% (*p* = 0.047) reduction in bone resorption.

### Longitudinal Evaluation of Bone Mass and Remodelling at the Tibia Midshaft

**Fig. 2:**
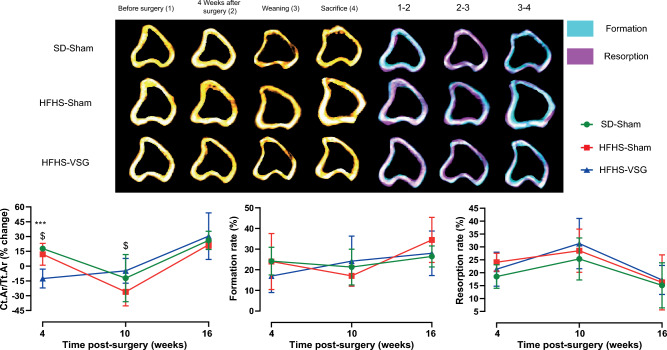
In vivo follow-up investigation of cortical bone mass and remodelling at the tibia midshaft. MicroCT images taken before surgery (1), 4 weeks after surgery (2), at weaning (3) and at sacrifice (4). Bone formation and resorption rates were computed before and 4 weeks after surgery (period 1–2), between 4 weeks post-surgery and weaning (period 2–3), and between weaning and sacrifice (period 3–4). Quantitative analysis of Ct.Ar/Tt.Ar is reported as % change from previous time point and not from baseline. Statistical differences between SD-Sham (*n* = 8), HFHS-Sham (*n* = 7) and HFHS-VSG (*n* = 10) were computed using a one-way ANOVA with Tukey multiple comparisons. ***: *p* < 0.0001 vs. SD-Sham and ^$^: *p* < 0.05 vs. HFHS-Sham at each time point

At the tibia midshaft, in the SD-Sham group, a significant augmentation (+ 18%, *p* = 0.037) of cortical bone mass, represented by Ct.Ar/Tt.Ar, was evidenced between pre- and post-surgery evaluations (Fig. 2). Ct.Ar/Tt.Ar was not further modified significantly at weaning but was augmented by 38% (*p* < 0.001) at sacrifice and associated with significant cortical resorption (−9%, *p* = 0.029). In the HFHS-Sham group, no significant alteration of Ct.Ar/Tt.Ar was evidenced between pre- and post-surgery period. However, at weaning these animals presented with a significant reduction in Ct.Ar/Tt.Ar (−14%, *p* = 0.016). At sacrifice, Ct.Ar/Tt.Ar was augmented by 47% (*p* = 0.005), as compared with weaning, and associated with significant augmentation of cortical formation (17%, *p* = 0.025), and reduction in cortical resorption (12%, *p* = 0.006). In the HFHS-VSG group, animals reduced significantly Ct.Ar/Tt.Ar by 13% (*p* = 0.002). At weaning, these animals did not present with a significant alteration of Ct.Ar/Tt.Ar (*p* = 0.37). However, at sacrifice, Ct.Ar/Tt.Ar was significantly augmented by 35% (*p* = 0.033) and associated with a significant reduction in cortical resorption (−22%, *p* = 0.027).

### Ex Vivo Evaluation of Trabecular Microarchitecture at the Proximal Tibia Metaphysis and Cortical Microarchitecture at the Tibia Midshaft

**Fig. 3:**
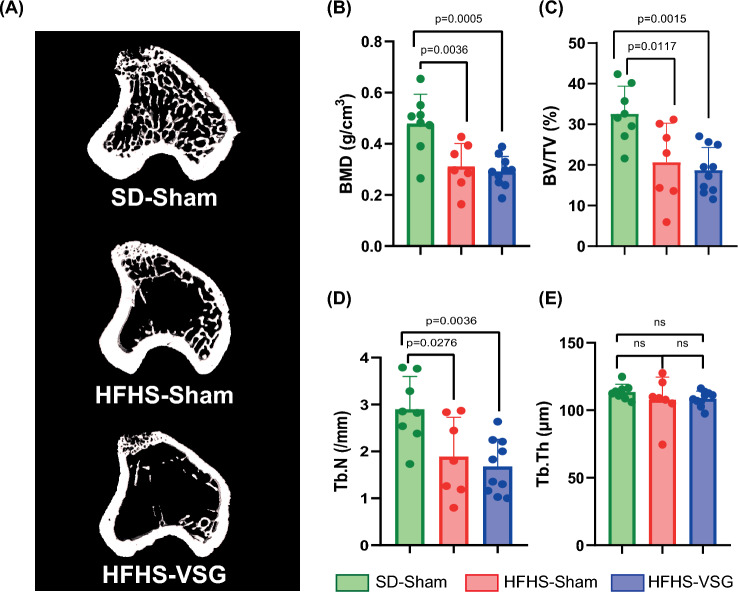
Ex vivo assessment of trabecular bone mass and microarchitecture at the proximal tibia metaphysis. **A** 3D model of each animal group. **B**–**E** BMD, BV/TV, Tb.N, Tb.Th were measured by microCT. Statistical differences between SD-Sham (*n* = 8), HFHS-Sham (*n* = 7) and HFHS-VSG (*n* = 10) were computed using a one-way ANOVA with Tukey multiple comparisons

After sacrifice, the tibia microarchitecture was analysed with a higher resolution as used for longitudinal evaluation of bone mass and remodelling. Figure 3A represents 3D-models of each animal group. As evidenced in Fig. 3B, trabecular bone mineral densities (BMD) were significantly reduced in HFHS-Sham (−35%) and HFHS-VSG (−39%) as compared with SD-Sham, respectively. However, no significant differences were noted between the two HFHS groups. These reductions of BMD were mirrored by reductions in BV/TV in HFHS-Sham (−36%) and HFHS-VSG (−42%) as compared with SD-Sham controls. Decreases in Tb.N, but not Tb.Th, were evidenced in HFHS-Sham (−34%) and HFHS-VSG (−41%) as compared with SD-Sham. Interestingly, the ex vivo BV/TV was significantly correlated with the BV/TV measured from the longitudinal assessment at sacrifice (R^2^ = 0.42, *p* < 0.001), validating the longitudinal follow-up.

**Fig. 4:**
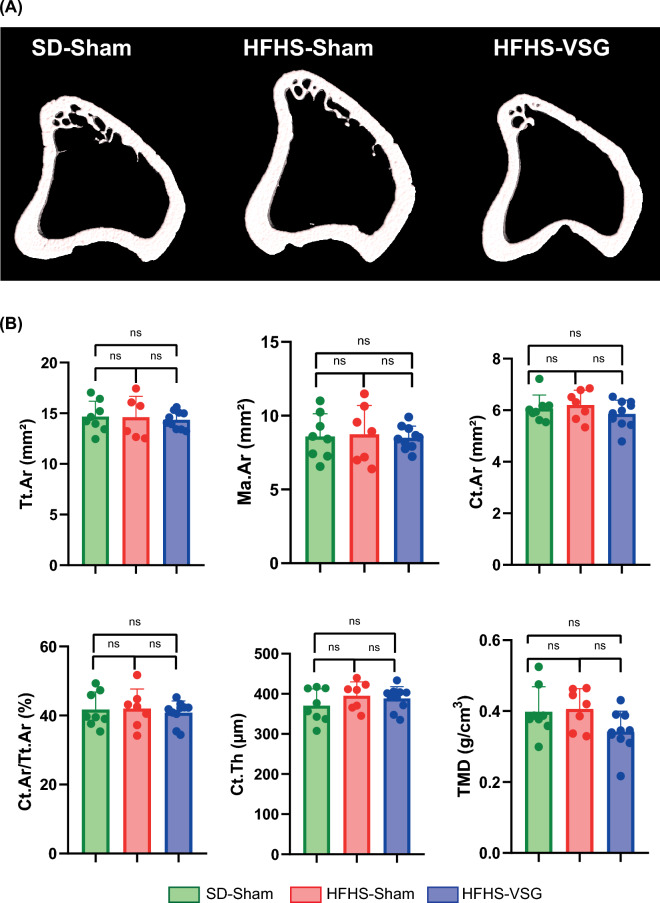
Ex vivo assessment of cortical microarchitecture at the tibia midshaft. **A** 3D model of each animal group. **B** Tt.Ar, Ma.Ar, Ct.Ar, Ct.Ar/Tt.Ar, Ct.th and TMD were measured by microCT. Statistical differences between SD-Sham (*n* = 8), HFHS-Sham (*n* = 7) and HFHS+VSG (*n* = 10) were computed using a one-way ANOVA with Tukey multiple comparisons

At the tibia midshaft, 3D models did not suggest a specific alteration of cortical microarchitecture (Fig. 4) and indeed, none of the conventional cortical microarchitecture parameters was significantly altered by HFHS or Sham/VSG surgeries. However, and in parallel to what was seen in the proximal metaphysis, Ct.Ar/Tt.Ar measured ex vivo was significantly correlated to the measure at sacrifice on the longitudinal evaluation (R^2^ = 0.42, *p* = 0.001).

### Evaluation of Biomechanical Response

As bariatric surgery is linked to excessive bone fragility, we performed 3-point bending measurement at the femur mid-diaphysis in all three groups. Animals under HFHS diet, albeit of the type of surgery, exhibited significant lower ultimate load (20–26%), yield load (30–32%), stiffness (19–23%) and work-to-fracture (18–20%) as compared with SD-Sham animals (Table 1). In order to ascertain whether changes in bone biomechanics were due to bone microarchitecture or changes in bone matrix biomechanics, we also computed tissue strength. Interestingly, tissue strength was significantly lower in HFHS-Sham (−21%, *p* = 0.008) and HFHS-VSG (−21%, *p* = 0.005) animals as compared with SD-Sham suggesting a possible alteration of bone material properties.

**Table 1:** Biomechanical parameters measured by 3-point bending at the midshaft femur

Biomechanical parameters	SD-Sham	HFHS-Sham	HFHS-VSG
Ultimate Load (N)	211 ± 15	169 ± 20^**^	156 ± 33^***^
Yield Load (N)	154 ± 19	108 ± 16^***^	104 ± 23^***^
Stiffness (N/mm)	669 ± 44	543 ± 90^*^	513 ± 85^***^
Post-yield displacement (mm)	0.52 ± 0.14	0.60 ± 0.15	0.67 ± 0.22
Work-to-fracture (N.mm)	118 ± 22	94 ± 13^*^	97 ± 19^*^
Peak Moment Bending (N.mm)	796 ± 60	634 ± 75^**^	602 ± 118^***^
Section Modulus (mm^3^)	3.192 ± 0.471	3.196 ± 0.393	3.044 ± 0.543
Tissue Strength (MPa)	252 ± 27	199 ± 33^**^	199 ± 30^**^

Statistical differences between SD-Sham (*n* = 8), HFHS-Sham (*n* = 7) and HFHS-VSG (*n* = 10) were investigated by one-way ANOVA with Tukey’s multiple comparison tests. ^*^: *p* < 0.05, ^**^: *p* < 0.001, ^***^: *p* < 0.0001 vs. SD-Sham

### Evaluation of Femur Microarchitecture and Geometry

**Fig. 5:**
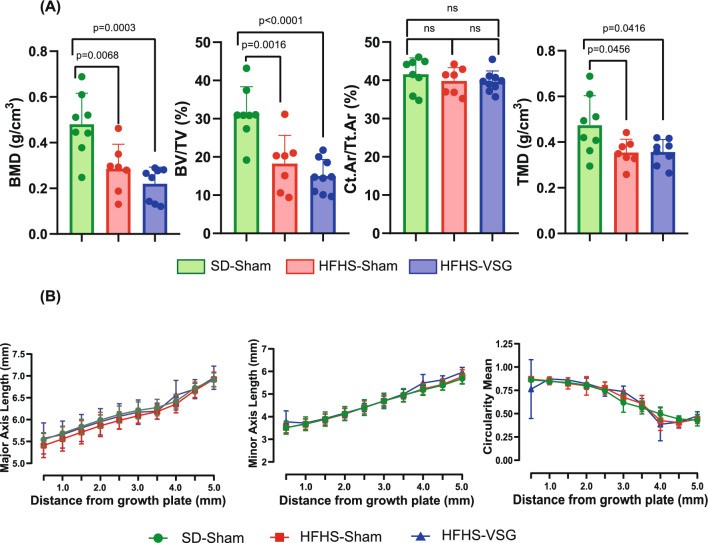
Ex vivo assessment of trabecular and cortical bone microarchitecture at the distal femur metaphysis and femur midshaft. **A** BMD, BV/TV, Ct.Ar/Tt.Ar and TMD were obtained by microCT. **B** Major axis length, minor axis length and circularity mean were measured from microCT scan. Statistical differences between SD-Sham (*n* = 8), HFHS-Sham (*n* = 7) and HFHS-VSG (*n* = 10) were computed using a one-way ANOVA with Tukey multiple comparisons

In order to ascertain whether alterations of bone biomechanics were due to specific alterations of bone microstructure at the femur that could not be observed at the tibia, we evaluated trabecular BMD and BV/TV at the distal femoral metaphysis (Fig. 5). Similar alterations as observed at the proximal tibia metaphysis were encountered and represented by lower BMD (−42% and −55%, *p* = 0.0068 and *p* = 0.0003) and BV/TV (−42% and −52%, *p* = 0.0016 and *p* < 0.0001) in HFHS-Sham and VSG groups, respectively. At the femoral diaphysis, as evidenced already at the tibia, no significant alterations of cortical microarchitecture could be evidenced between groups. However, tissue mineral density (TMD) was significantly lower in HFHS groups (−25% and −25%, *p* = 0.045 and *p* = 0.041) as compared with SD-Sham. No alterations of femur geometry were observed between groups. On the other hand, TMD was positively correlated with yield load (R^2^ = 0.20, *p* = 0.028).

### Evaluation of Bone Material Properties at the Femur Mid-Diaphysis

We then investigated the extracellular matrix properties by Raman spectroscopy on transaxial section of the mid-femur (Table 2). At the bone formation site, no significant differences between all three groups of animals were encountered. Interestingly, no alterations of the v1PO_4_/Amide I ratio, indicative of the degree of mineralization, was evidenced. We also examined material properties of the bone matrix by performing Raman spectroscopy over the full cortical width. In all animals, each parameter followed a Gaussian distribution. As such the mean and full width at half maximum (FWHM) were computed. Matrix nanoporosity was significantly lower in HFHS-VSG animals (−50%, *p* = 0.040) as compared to HFHS-Sham. Interestingly, the FWHM of v1PO_4_/Amide I was significantly lowered in HFHS-Sham (−53%, *p* = 0.035) and HFHS-VSG (−54%, *p* = 0.012) as compared with SD-Sham.

**Table 2:** Bone material properties of the femur midshaft measured by Raman microspectroscopy

Bone material parameters	SD-Sham	HFHS-Sham	HFHS-VSG
At bone formation site			
v_1_PO_4_/Amide I	10.83 ± 2.9	11.83 ± 2.7	10.02 ± 3.1
v_3_CO_3_/v_1_PO_4_	0.164 ± 0.006	0.165 ± 0.009	0.162 ± 0.010
Nanoporosity	73 ± 30.3	61.6 ± 44.4	121.1 ± 85.4
Mineral crystallinity	0.106 ± 0.01	0.110 ± 0.008	0.108 ± 0.009
Proline hydroxylation content	0.62 ± 0.047	0.64 ± 0.05	0.69 ± 0.08
GAG/Amide III	0.0863 ± 0.006	0.0936 ± 0.016	0.0053 ± 0.015
1670 cm^−1^/1690 cm^−1^	2.739 ± 0.59	2.996 ± 0.76	2.233 ± 0.37
At full cortical width			
Mean v_1_PO_4_/Amide I	11.18 ± 1.0	11.93 ± 0.75	11.88 ± 1.11
Mean v_3_CO_3_/v_1_PO_4_	0.183 ± 0.004	0.178 ± 0.003	0.180 ± 0.005
Mean Nanoporosity	*0.0037* ± *0.0015*	*0.0042* ± *0.0027*	*0.0020* ± *0.0007*^*#*^
Mean Mineral crystallinity	0.055 ± 0.0002	0.055 ± 0.0002	0.0550 ± 0.0003
Mean Proline hydroxylation content	0.76 ± 0.02	0.74 ± 0.01	0.75 ± 0.02
Mean GAG/Amide III	0.475 ± 0.015	0.443 ± 0.014	0.454 ± 0.014
Mean 1670 cm^−1^/1690 cm^−1^	2.773 ± 0.079	2.802 ± 0.25	2.732 ± 0.093
FWHM v_1_PO_4_/Amide I	4.1 ± 2.1	*2.0* ± 0.7*	*1.9* ± 0.6*
FWHM v_3_CO_3_/v_1_PO_4_	0.014 ± 0.002	0.012 ± 0.006	0.012 ± 0.006
FWHM Nanoporosity	0.003 ± 0.001	0.003 ± 0.001	0.003 ± 0.001
FWHM Mineral crystallinity	0.0014 ± 0.0005	0.0013 ± 0.0003	0.0013 ± 0.0002
FWHM Proline hydroxylation content	0.15 ± 0.04	0.17 ± 0.01	0.13 ± 0.05
FWHM GAG/Amide III	0.123 ± 0.017	0.137 ± 0.008	0.125 ± 0.021
FWHM 1670 cm^−1^/1690 cm^−1^	0.2290 ± 0.017	0.2148 ± 0.006	0.2177 ± 0.006

Bone material properties were measured either at bone formation site by placing 3 points between double calcein labels or by drawing a line over the full cortical width. The latter allows for computation of parameter distribution, represented by a Gaussian shape, and reported as mean and full width at half maximum (FWHM). Statistical differences between SD-Sham (*n* = 8), HFHS-Sham (*n* = 7) and HFHS-VSG (*n* = 10) were computed using a one-way ANOVA with Tukey multiple comparisons. *: *p* < 0.05 vs. SD-Sham and ^#^: *p* < 0.05 vs. HFHS-Sham

The FWHM of v1PO_4_/Amide I was positively correlated with the peak moment bending (R^2^ = 0.22, *p* = 0.022). On the other hand, nanoporosity, that was significantly altered in HFHS-VSG as compared with HFHS-Sham, was not correlated with peak moment bending (R^2^ = 0.03, *p* = 0.48).

## Discussion

Bariatric surgery has been associated with an increased bone fragility with a mechanism that remains to be fully elucidated. As bariatric surgery and VSG is increasingly performed in young obese women in order to restore fertility, we thought to ascertain whether a gestation followed by a lactation period could affect the bone fragility observed after VSG. To answer this question we used a rat model of pregnancy after VSG. In this study, we evidenced, as others [18, 20, 36, 37], that VSG resulted in rapid trabecular and cortical bone losses at the appendicular skeleton due to a significant increase in bone resorption. However, the gestation/lactation period seems to revert the bone phenotype with an increase in trabecular and cortical bone mass from end of lactation period until sacrifice in the investigated model. Interestingly, we also evidenced that bone fragility was present in HFHS animals independently of the type of surgery (Sham or VSG) and associated to lower tissue strength and alterations of mineralization heterogeneity. However, matrix nanoporosity was only affected in HFHS-VSG. This suggested that the diet and perturbations of lipid and carbohydrate metabolisms might be responsible of the bone fragility and alterations of material properties rather than the VSG surgery per se in the investigated rat model.

The bone loss due to calcium mobilization during gestation and lactation is more significant in a rodent model because litter size is larger. In rats, there is typically a reduction in bone mass of 15 to 35% at the spine and hip, respectively, accompanied by a decrease in trabecular and cortical thickness and a reduction in the number of bone trabeculae [38]. Nevertheless, whether in female individuals or rodents, and even though the exact mechanisms are not fully understood, an increase in osteoclast resorption during gestation/lactation period followed by an increase in the population of active osteoblasts at the end of lactation has been highlighted [39]. This could explain the recovery of trabecular bone mobilized during lactation and observed in our study, especially with respect to the higher bone formation observed in the longitudinal follow-up. Although not investigated in the present study, the augmentation in active osteoblasts following the lactation period may involves several endocrine mediators such as estradiol, prolactin, vitamin D, parathyroid hormone, parathyroid hormone-related peptide, calcitonin, and gonadotrophins [38–41].

Obesity is associated with increases in BMI and BMD [42] despite a higher risk of fractures in obese children, adolescents, and adults [3, 43]. It is important to ascertain whether rodent models of obesity could recapitulate the findings observed in humans. Several animal studies have showed that a high-fat diet leads to bone repercussions, including a decrease in BV/TV and Tb.N in long bones [44, 45] despite higher body weight. As such the trabecular bone phenotype in rodent is not identical as the one seen in humans. Nevertheless, in the present study, we reported alterations of trabecular bone mass in animals under a HFHS diet and after pregnancy/lactation periods that are similar to previously reported in mature and immature rodents [44, 45]. Interestingly, although fertility and energy metabolism have been ameliorated following bariatric surgery [46–51], it seems that bone alterations due to HFHS diet are unaffected by VSG in the investigated model.

On the other hand, and as hypothesized as the beginning of this study, it seems that pregnancy and lactation did not aggravate the bone phenotype due to VSG in our model. More importantly, changes in patterns of bone resorption and bone formation following these periods appeared similar between HFHS-Sham and HFHS-VSG suggesting that bariatric surgery is not detrimental to bone remodelling programming in this model. However, despite similar cortical geometry and microarchitecture at the femur, HFHS animals exhibited a bone fragility that seems to be associated with the quality of the extracellular matrix, especially with the lack of heterogeneity in the degree of mineralization. This alteration has previously been observed in atypical fracture in women with bisphosphonate treatment [52, 53]. A plausible scenario is represented by adipokine contribution. Indeed, adipose tissue is known to release pro-inflammatory cytokines (IL-6, TNF-α, IL-1β) that reduce osteoblastic differentiation of mesenchymal stem cells, decrease bone formation [54] allowing more time for the matrix to mineralised and reducing heterogeneity.

A strength of our study is represented by the longitudinal follow-up of bone mass and bone remodelling that allowed us to precisely quantify 3D bone remodelling and trabecular and cortical bone microarchitecture at different time points, which, to our knowledge, has never been done in a rat model before. Without this follow-up, we would have overlooked the bone loss induced by VSG, as animals on the HFHS diet appear identical at terminal endpoints in terms of trabecular and cortical bone. Another strength relies in the full assessment of trabecular and cortical bone mass, microarchitecture, biomechanics and material properties that allow to evidence that the alteration of bone biomechanics is due to changes in matrix properties rather than changes in bone microarchitecture. A limitation of this study is represented by the model organism that does not reproduce the trabecular bone phenotype observed in human obese individuals, especially in term of higher trabecular BMD due to higher body weight. Although the structure of the cortical bone is lamellar in rodents, it does not present a haversian structure and intracortical bone remodelling. Nevertheless, similar trabecular bone loss at the tibia proximal metaphysis was observed rapidly after VSG, similar to what is observed after bariatric surgery in human individuals [55].

## Conclusions

In our study, we demonstrated that pregnancy and lactation periods after vertical sleeve gastrectomy did not aggravate the bone fragility in a rodent model. However, this study suggests that bone fragility due to high fat high sugar diet, represented by modifications of material properties, is not reversed by vertical sleeve gastrectomy, at least at cortical bone site. Further investigation in humans is required to test the potential benefits of bariatric surgery on obesity-induced bone fragility.

## Supplementary Information

Below is the link to the electronic supplementary material.

Supplementary file1 (PDF 191 kb)

## Data Availability

The data that support the findings of this study are available from the corresponding author upon reasonable request.
