# Supplementary material for: Effects of Pregnancy and Lactation on Bone Microstructure and Material Properties in a Rat Model of Bariatric Surgery
Source: Calcif Tissue Int. 2025 Jan 4;116(1):23. doi: 10.1007/s00223-024-01321-1 (PMC11700058; doi:10.1007/s00223-024-01321-1)

Supplemental material

Supplementary figure 1: Schematic of the study design

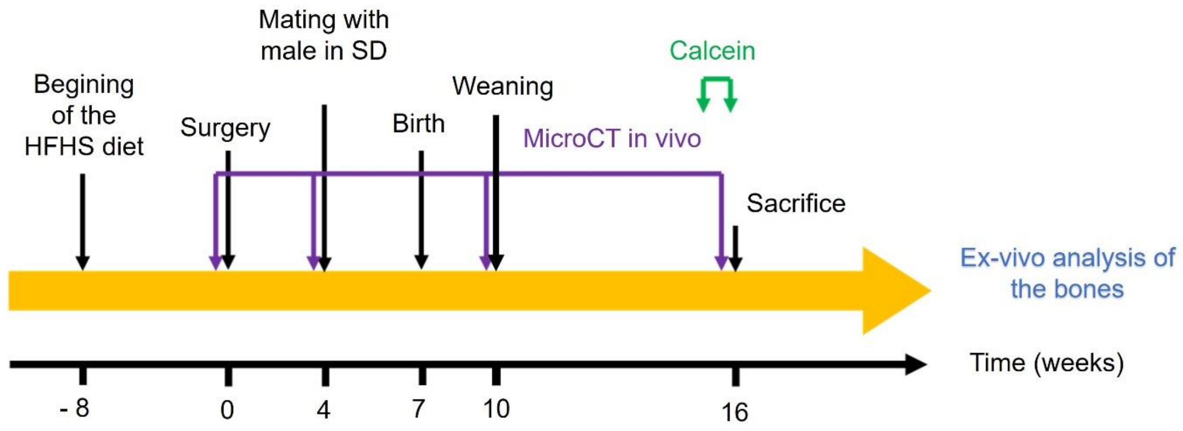

Supplementary figure 2: Effects of standard (SD) and high-fat high-sugar (HFHS) diet on body weight and abdominal adipose tissue at 14 weeks of age.

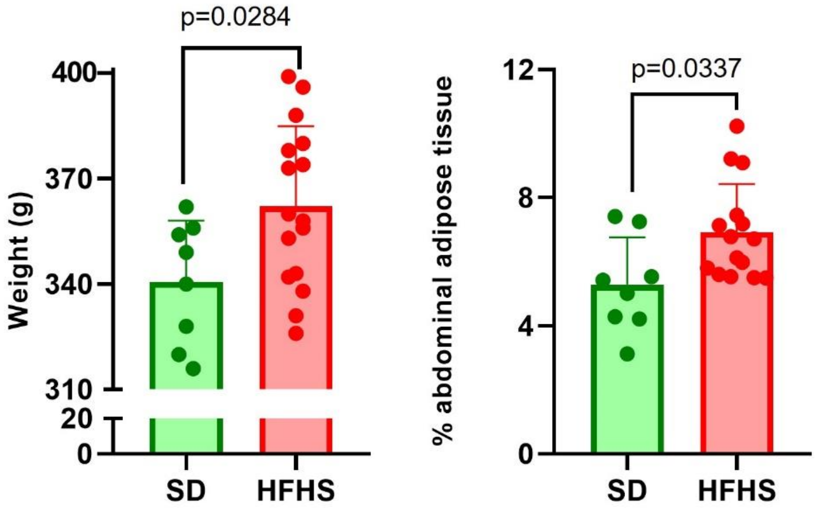

Supplement: Supplementary file 1 — Supplementary file1 (PDF 191 kb) [file 223_2024_1321_MOESM1_ESM.pdf]
